# Digital Implant Rehabilitation in Multiple Idiopathic Root Resorption: A Case Report

**DOI:** 10.1002/ccr3.73421

**Published:** 2026-08-27

**Authors:** Shoresh Afnan, Thomas Alexander Cursiter Hunter, Kåre Jan Attramadal, Jashan Singh Kamboj

**Affiliations:** ^1^ Oris Academy Oslo Norway; ^2^ Oris Dental Oslo Norway

**Keywords:** dental implants, digital dentistry, digital planning, guided surgery, implant bridge, multiple idiopathic root resorption

## Abstract

Multiple idiopathic root resorptions (MIRs) represent a rare condition with uncertain etiology and limited treatment options, and they may compromise the prognosis of affected teeth. A digitally guided interdisciplinary approach combining cone‐beam computed tomography (CBCT) diagnostics, virtual implant planning, and immediate implant‐supported restoration can provide predictable, functional, and esthetic outcomes in complex cases.

**Abbreviation**
MIR: multiple idiopathic root resorptions

## 1 Introduction

Root resorption presents a challenge for both patients and clinicians, as early diagnosis is critical for prognosis and for preserving the affected dentition. Diagnosis is based on the location and etiological factors of the resorptive process. A simplified classification system proposed by Darcey distinguishes between internal and external root resorption [1, 2].

Internal root resorption requires the presence of both vital tissue in the resorptive area and necrotic or infected pulpal tissue coronally. When detected at an early stage, endodontic and restorative management may preserve affected teeth and remain the preferred treatment approach [1, 2, 3].

External root resorption is categorized as infection‐related resorption, external cervical resorption, and replacement resorption. External infection‐related resorptions most commonly occur following dental trauma and require close follow‐up. Early diagnosis is essential to prevent significant loss of tooth substance or bone volume [1, 2, 3, 4].

Multiple idiopathic root resorption (MIR) describes a condition characterized by significant damage to root surfaces with an idiopathic etiology [3, 4]. Certain systemic and rare genetic disorders, including hypoparathyroidism, hyperparathyroidism, calcinosis, Turner syndrome, Gaucher's disease, and Paget's disease, have been associated with multiple idiopathic resorptive lesions and should therefore be considered and excluded during diagnostic assessment [5]. Diagnosis is primarily performed using cone‐beam computed tomography (CBCT) imaging, as the resorptive lesions lie below the epithelial attachment and often beneath the bone level [4]. CBCT‐based assessments have been shown to reliably quantify apical root resorption and alveolar bone height, supporting its central role in diagnosis and treatment planning [6].

The condition is mostly asymptomatic until pulp involvement occurs [4]. Treatment aims to remove the resorptive tissue, which generally requires surgical access to expose the lesion. Surgical exposure and bone removal are frequently necessary. Treatment of such cases typically requires interdisciplinary collaboration among endodontists, oral surgeons, and prosthodontists [2, 7]. Modern technologies such as digital workflows and virtual planning tools are essential to ensure precision and efficiency throughout the treatment process [8, 9, 10].

This case report describes the treatment of a 50‐year‐old male patient with no significant medical history, no history of smoking or periodontal disease, no current medication use, and no known allergies. He was referred because of extensive resorption affecting the maxillary incisors. Following a comprehensive clinical and radiological evaluation, a diagnosis of MIR was confirmed. Due to irreversible root damage, endodontic treatment was not feasible. In accordance with the patient's wish for a highly esthetic and functional restoration, a digitally guided implant‐supported rehabilitation was planned and carried out.

This report highlights the integration of CBCT, intraoral scanning, and virtual implant planning with an immediate loading protocol to support precise treatment execution. It also underscores the importance of interdisciplinary collaboration and a patient‐centered approach in achieving favorable esthetic and functional outcomes.

## 2 Case Presentation

**FIGURE 1:**
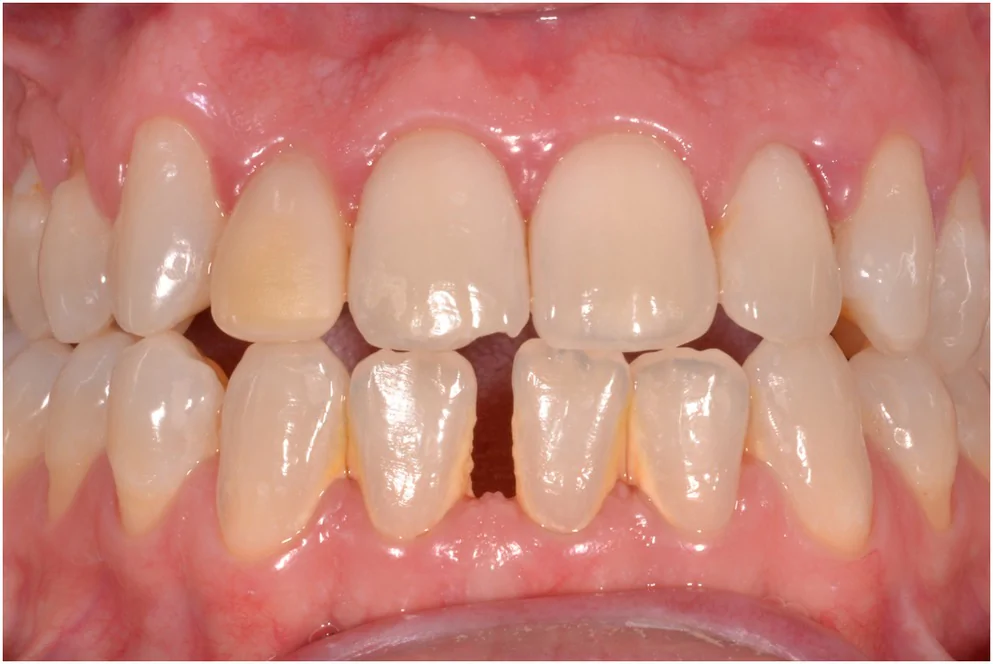
Frontal intraoral photograph showing the patient's occlusion in maximum intercuspation before treatment, taken on 30.10.2017.

**FIGURE 2:**
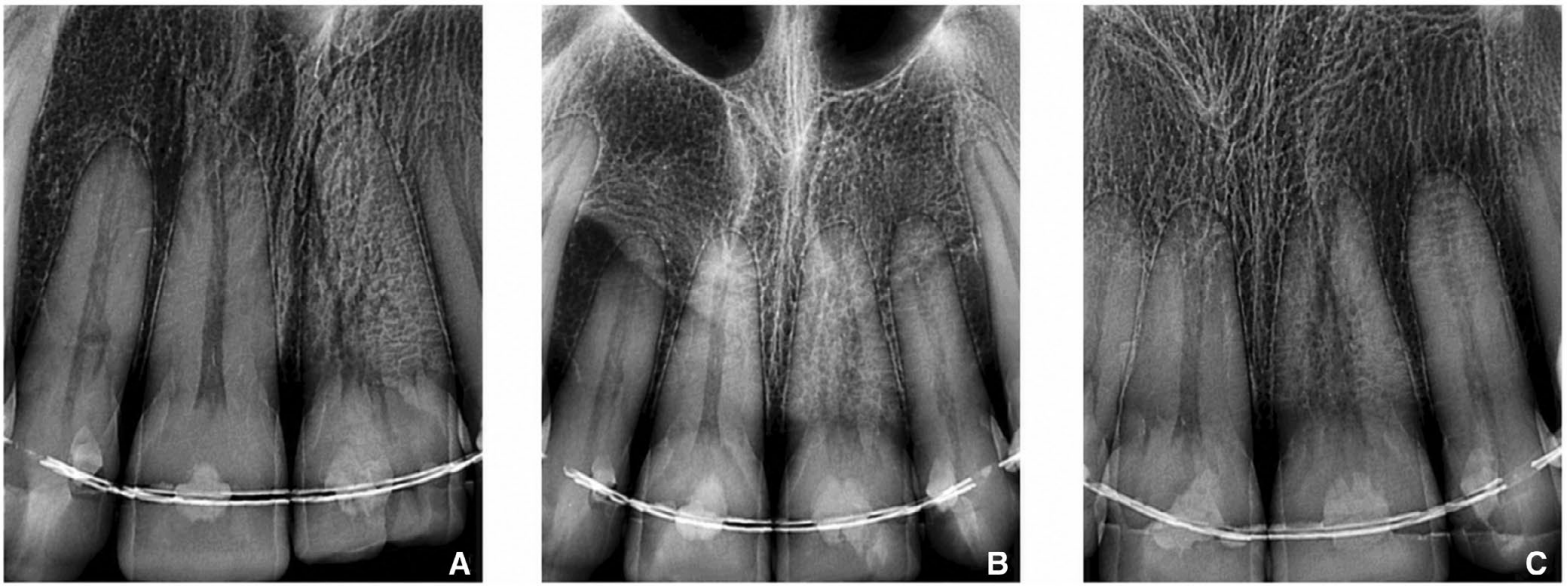
Preoperative intraoral radiographic examination of the maxillary anterior region, obtained on October 30, 2017. (A) Lateral intraoral radiograph of the left maxillary anterior region. (B) Intraoral radiograph of the maxillary anterior region. (C) Lateral intraoral radiograph of the right maxillary anterior region.

A 50‐year‐old male patient (Figure 1) was referred to an endodontic specialist at Oris Dental Galleri Oslo by his general dentist because of radiological findings. Radiographs revealed radiolucent areas around the roots of the maxillary incisors, raising suspicion of multiple resorptions (Figure 2A–C).

The patient reported mobility and looseness of the upper incisors. Clinical examination revealed no pathological changes on the supragingival surfaces or in the periodontal pockets.

Vitality testing, including electric pulp testing (EPT) and cold sensitivity testing, demonstrated no response in Teeth 12 through 22. The patient also reported having undergone previous orthodontic treatment and wearing a fixed maxillary retainer for approximately 20 years. Although previous orthodontic treatment and the long‐term presence of the retainer could not be excluded as potential contributing factors, root resorption was also present in the mandibular dentition.

A CBCT scan was requested and evaluated by a specialist in maxillofacial radiology. CBCT imaging was performed using the Veraviewepocs 3D R100 with a voxel size of 0.125 mm. The original CBCT dataset had been deleted prior to preparation of this report. Therefore, the specific imaging parameters could not be retrieved from the system records. The CBCT scan was obtained to evaluate the extent of the lesion and the remaining hard tissue structures in the anterior maxillary region. Radiographic assessment demonstrated lesion involvement associated with Teeth 12, 11, 21, and 22, with Tooth 11 classified as Heithersay Grade 3, Tooth 12 as Heithersay Grade 3–4, Tooth 21 as Heithersay Grade 4, and Tooth 22 as Heithersay Grade 3. No change in the overall length of the affected teeth was detected. The radiological report confirmed multiple resorptions in the incisors of both the maxilla and mandible, providing a comprehensive overview of their extent and location.

**FIGURE 3:**
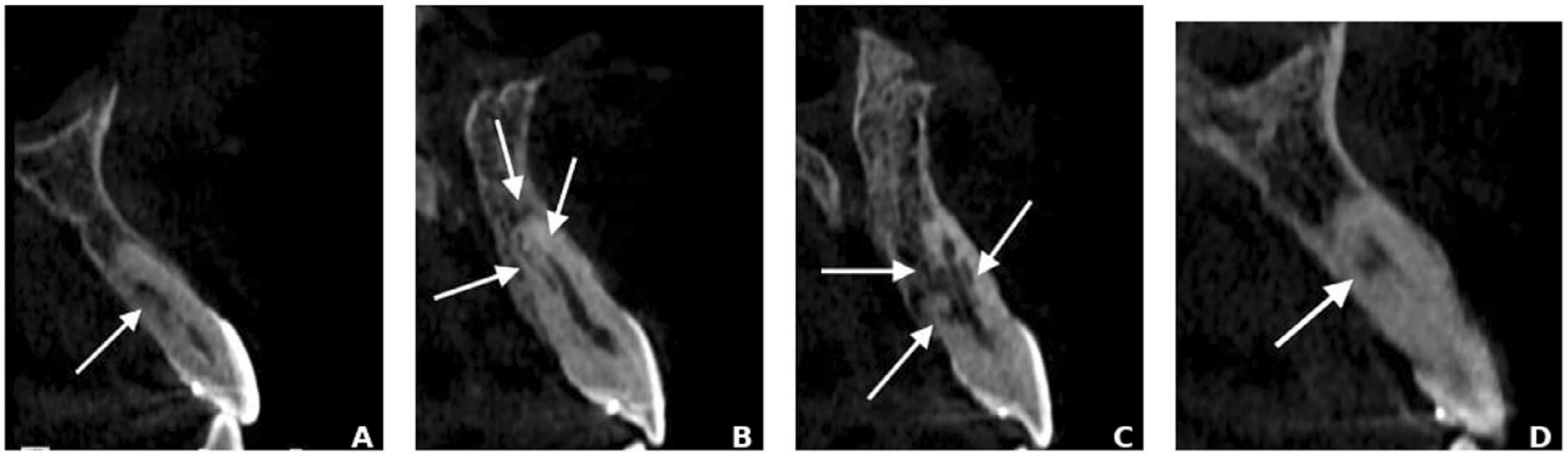
CBCT sagittal sections of the maxillary incisors, obtained on December 11, 2017. (A) Sagittal section of a maxillary incisor. (B) Sagittal section of a maxillary incisor, with arrows indicating the area of resorption. (C) Sagittal section of a maxillary incisor, with arrows indicating the area of resorption. (D) Sagittal section of a maxillary incisor, with arrows indicating the area of resorption.

Based on the clinical and radiological findings, a diagnosis of idiopathic multiple irreversible resorptions (MIR) was confirmed. Due to severe irreversible damage to the apical portions of the roots, the prognosis for endodontic treatment was deemed poor (Figure 3A–D). The case was presented at the clinic's weekly specialist meeting. A general dentist with formal training in implant prosthetics was included in the subsequent planning. Consensus was reached across disciplines. Together with the patient's general dentist, prosthodontist, and specialist in oral surgery and medicine, the patient was informed of the proposed treatment: extraction of Teeth 12, 11, 21, and 22 followed by implant‐supported bridge restoration.

The patient was internally referred to the Oral Surgery and Medicine Department at Oris Dental Galleri Oslo for further treatment.

## 3 Methods

During the initial consultation, the patient's wishes, expectations, and goals were assessed. The patient desired high esthetic quality, optimal function, and an efficient treatment process. Removable prosthetics were excluded. A conventional prosthetic bridge solution was deemed inappropriate because of an otherwise intact dentition.

**FIGURE 4:**
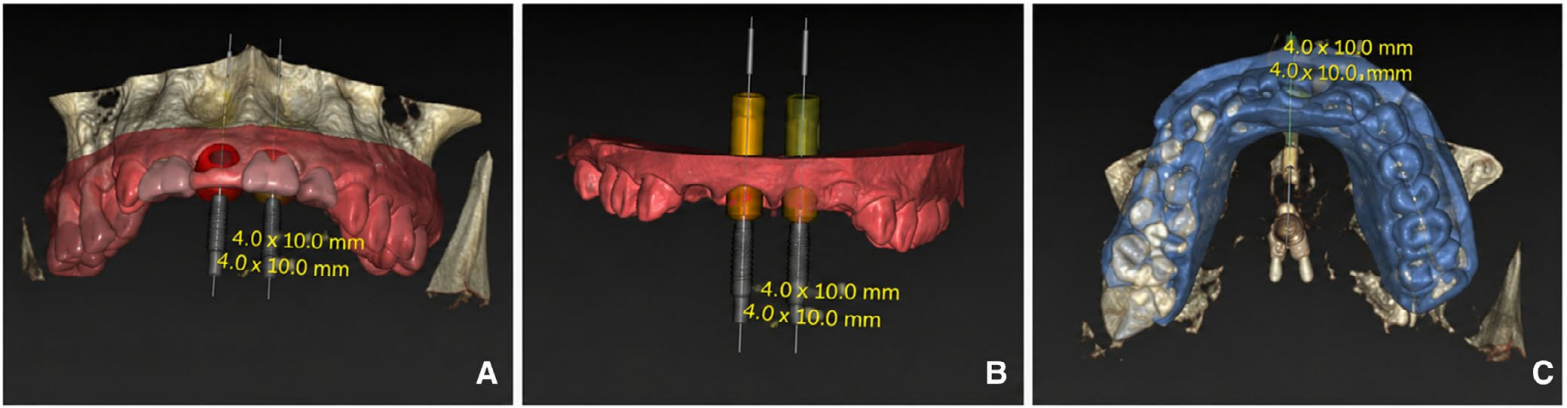
Screenshots from DTX Studio illustrating the digital implant planning process, obtained on June 22, 2018. (A) Digital planning process. (B) Planned angulation and insertion direction of the implants. (C) Digital mock‐up of the final restoration.

**FIGURE 5:**
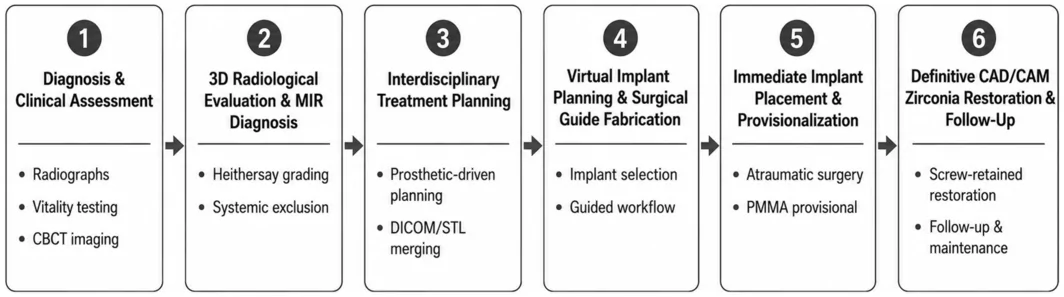
A structured six‐step workflow beginning with referral and clinical examination, followed by CBCT‐based radiological assessment. Diagnostic and interdisciplinary treatment planning guide the transition to digital integration of CBCT (DICOM) and intraoral scan (STL) data. Virtual prosthetically driven implant planning is performed in dedicated software, enabling guided surgical execution and immediate provisionalization. The workflow concludes with definitive restoration and structured clinical follow‐up.

Following interdisciplinary evaluation and consultation with the patient and the referring general dentist, implant therapy was selected. A digital workflow combined with an immediate loading protocol was planned. Table 1 provides an overview of the chronological sequence of clinical events and their corresponding figures. Treatment followed a prosthetically guided implant placement concept, and a screw‐retained solution was recommended (Figure 4A–C). Figure 5 illustrates the digitally guided workflow used in this case, from diagnostic assessment and CBCT‐based evaluation to prosthetically driven implant planning, guided surgery, immediate provisionalization, and definitive CAD/CAM prosthetic rehabilitation.

**TABLE 1:** Clinical timeline of the surgical procedure within a fully digital workflow, from initial examination to final treatment completion.

Date	Clinical stage	Corresponding figures
30.10.2017	Initial clinical examination	Figure 1
30.10.2017	Initial radiographic assessment	Figure 2A–C
11.12.2017	CBCT examination and diagnostic confirmation	Figure 3A–D
22.06.2018	Digital treatment planning	Figure 4A–C
22.06.2018	Emergence profile and digital prosthetic workflow	Figure 7A
25.01.2019	Surgical treatment and immediate implant placement	Figure 6A–E,H
Feb. 2019	Provisional and laboratory prosthetic fabrication	Figures 6F,G and 7C,D
23.05.2019	Definitive prosthetic delivery	Figures 6G, 7B, and 8A,B
16.12.2020	Follow‐up evaluation	Figure 9B
25.04.2022	Long‐term follow‐up	Figure 9C
30.01.2023	Long‐term follow‐up	Figure 9D
16.01.2024	Long‐term follow‐up	Figure 9E
24.02.2025	Final follow‐up evaluation	Figure 9F

DICOM files from the CBCT scans and STL files from the intraoral scans were merged in DTX Studio (Nobel Biocare) [9] to enable prosthetically driven virtual implant planning and restorative design. Direct intraoral scanning generates three‐dimensional models with dimensional accuracy comparable to that of extraoral scanning of conventional stone casts, thereby supporting reliable prosthetic planning [11]. Based on the digital plan, implants were placed in regions 11 and 21, with pontics in regions 12 and 22. This decision was based on:

- Implant angulation in the lateral regions would compromise esthetics and require a cement‐retained prosthesis.
- Greater bone volume and soft tissue quality in regions 11 and 21.
- Better biomechanical load distribution in the central area.

**FIGURE 6:**
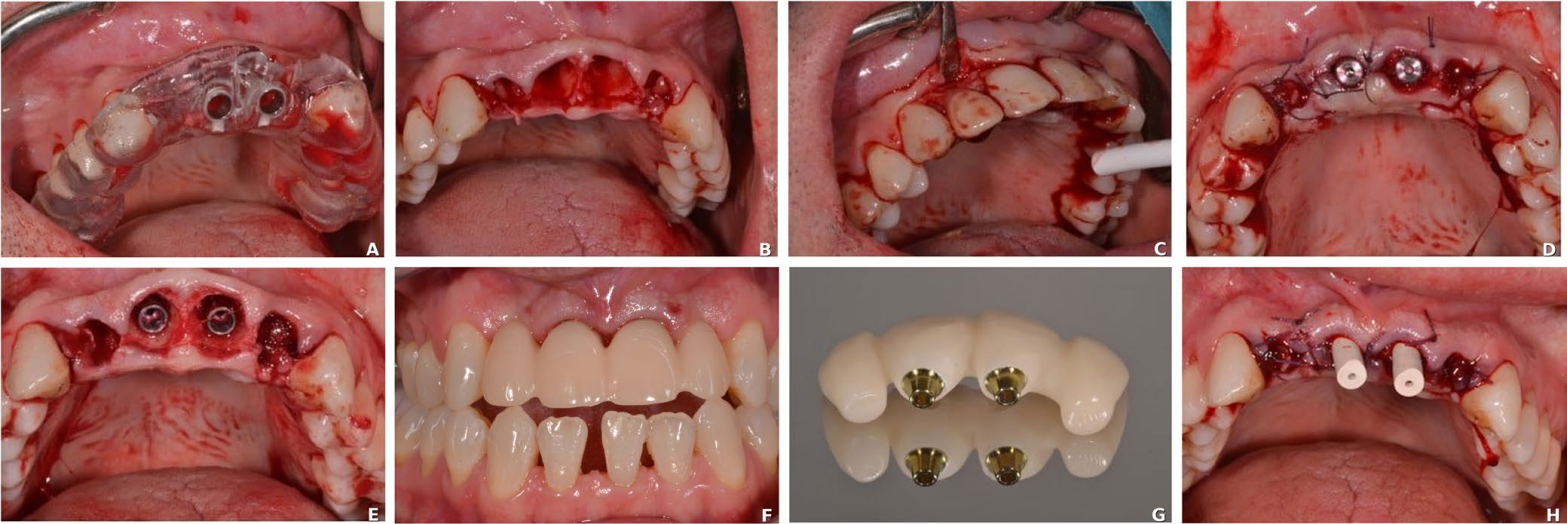
Perioperative, postoperative, and prosthetic clinical documentation of the implant rehabilitation. (A) Perioperative occlusal intraoral photograph showing the surgical stent in position from a lateral view, obtained on January 25, 2019. (B) Perioperative occlusal intraoral photograph after extraction, obtained on January 25, 2019. (C) Perioperative occlusal intraoral photograph before extraction, obtained on January 25, 2019. (D) Postoperative frontal intraoral photograph, obtained on January 25, 2019. (E) Perioperative occlusal intraoral photograph, obtained on January 25, 2019. (F) Frontal intraoral photograph showing the patient's occlusion in centric relation, obtained on January 25, 2019. (G) Clinical photograph of the ceramic bridge, obtained in February 2019. (H) Intraoral photograph showing scan bodies in place after implant insertion, obtained on January 25, 2019.

A surgical guide was digitally fabricated using DTX Studio [9] (Figure 6A). Preoperatively, the patient received Dalacin 300 mg twice daily and ibuprofen 600 mg twice daily, and local anesthesia was achieved using xylocaine/adrenaline 20 mg/mL + 12.5 μg/mL. A minimally invasive approach was used, with a limited incision performed only to relieve the interdental papilla and allow optimal implant positioning. A flapless technique was otherwise maintained to preserve soft tissue architecture and vascular supply (Figure 6B,C). Two implants (Nobel Parallel, 3.75 × 15 mm, Nobel Biocare) were placed atraumatically immediately after extraction in the positions of Teeth 11 and 21 using a prosthetically guided approach (Figure 6D,E). Implant site preparation was carried out according to the manufacturer's drilling protocol for medium‐density bone (Type II–III) for NobelParallel Conical Connection implants [12]. Sequential osteotomy preparation was performed using a pilot drill (2.0 mm), followed by a twist drill (2.4/2.8 mm), both to full depth (15 mm), under copious external irrigation.

An undersized osteotomy was intentionally maintained in accordance with the manufacturer's recommendations to achieve optimal primary stability. Drilling was performed using intermittent motion with continuous irrigation to minimize thermal injury [12].

The protocol was designed to achieve an insertion torque between 35 and 45 Ncm, thereby supporting immediate loading. Adequate primary stability was achieved, allowing immediate loading with a provisional restoration. Following implant placement, implant‐supported provisionalization was performed using prefabricated dynamic abutments (Dynamic Abutment CC, Nobel Biocare). A screw‐retained provisional bridge was fabricated from printed Tempshell PMMA in shade A1 and adjusted to achieve optimal occlusion and esthetics (Figure 6F,G). The provisional restoration was torqued to 15 Ncm. The patient received Apocillin 660 mg four times daily for 5 days to reduce the risk of postoperative complications, in accordance with the clinic's guidelines. Postoperative instructions included daily rinsing with a 2% chlorhexidine solution and a soft‐food diet for 2 weeks to reduce the risk of infection and maintain favorable healing conditions.

**FIGURE 7:**
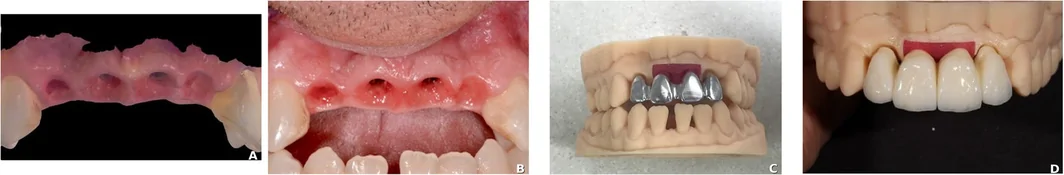
Digital and clinical documentation of the definitive prosthetic rehabilitation. (A) Screenshot from DTX Studio showing the intraoral scan after establishment of the emergence profile, obtained on June 22, 2018. (B) Clinical intraoral photograph at follow‐up before insertion of the definitive bridge, obtained on May 23, 2019. (C) Clinical photograph of the metal framework of the bridge before ceramic veneering, obtained in February 2019. (D) Clinical photograph of the final bridge after ceramic veneering, obtained in February 2019.

Digital impressions were obtained using an intraoral scanner (3Shape), allowing for precise prosthetic planning and fabrication (Figures 6H and 7A–D).

The patient reported no postoperative complications.

## 4 Outcome and Follow‐Up

**FIGURE 8:**
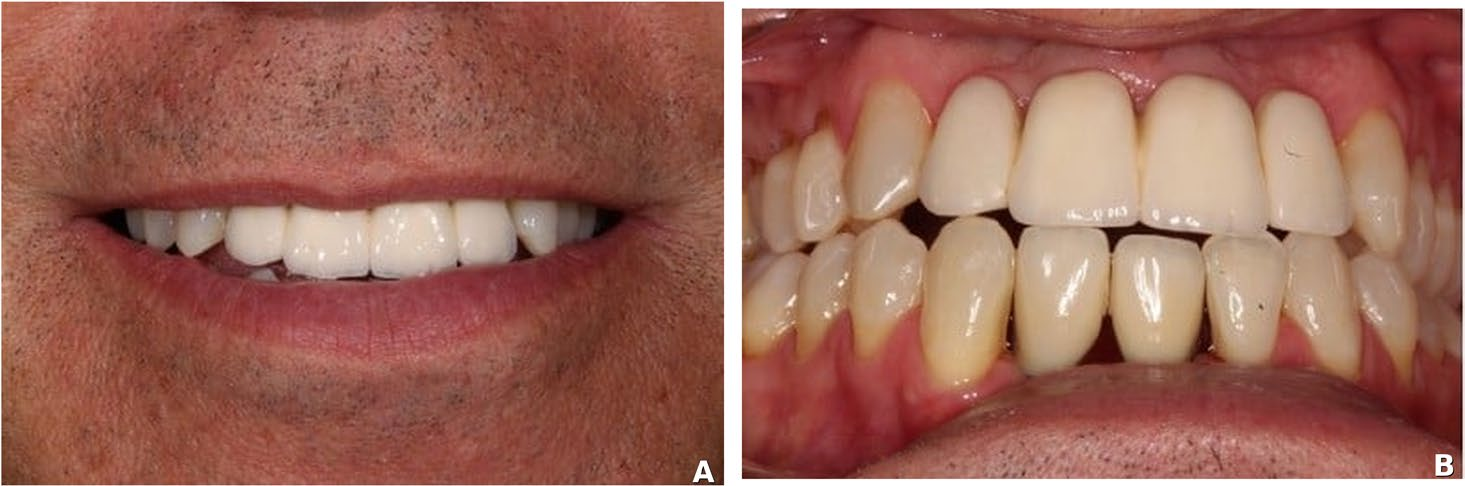
Clinical outcome following completion of treatment, documented on May 23, 2019. (A) Extraoral frontal photograph of the patient after completion of treatment. (B) Frontal intraoral photograph showing the patient's occlusion in maximum intercuspation after treatment.

After a healing period of 3 months, definitive prosthetic rehabilitation was completed using a screw‐retained zirconia bridge with a cutback design (Figure 8A,B). The final restoration was torqued to 35 Ncm using a dynamic abutment driver. Teflon tape was applied to seal the screw access channels, followed by composite restoration (Filtek Z250, shade A3).

**FIGURE 9:**
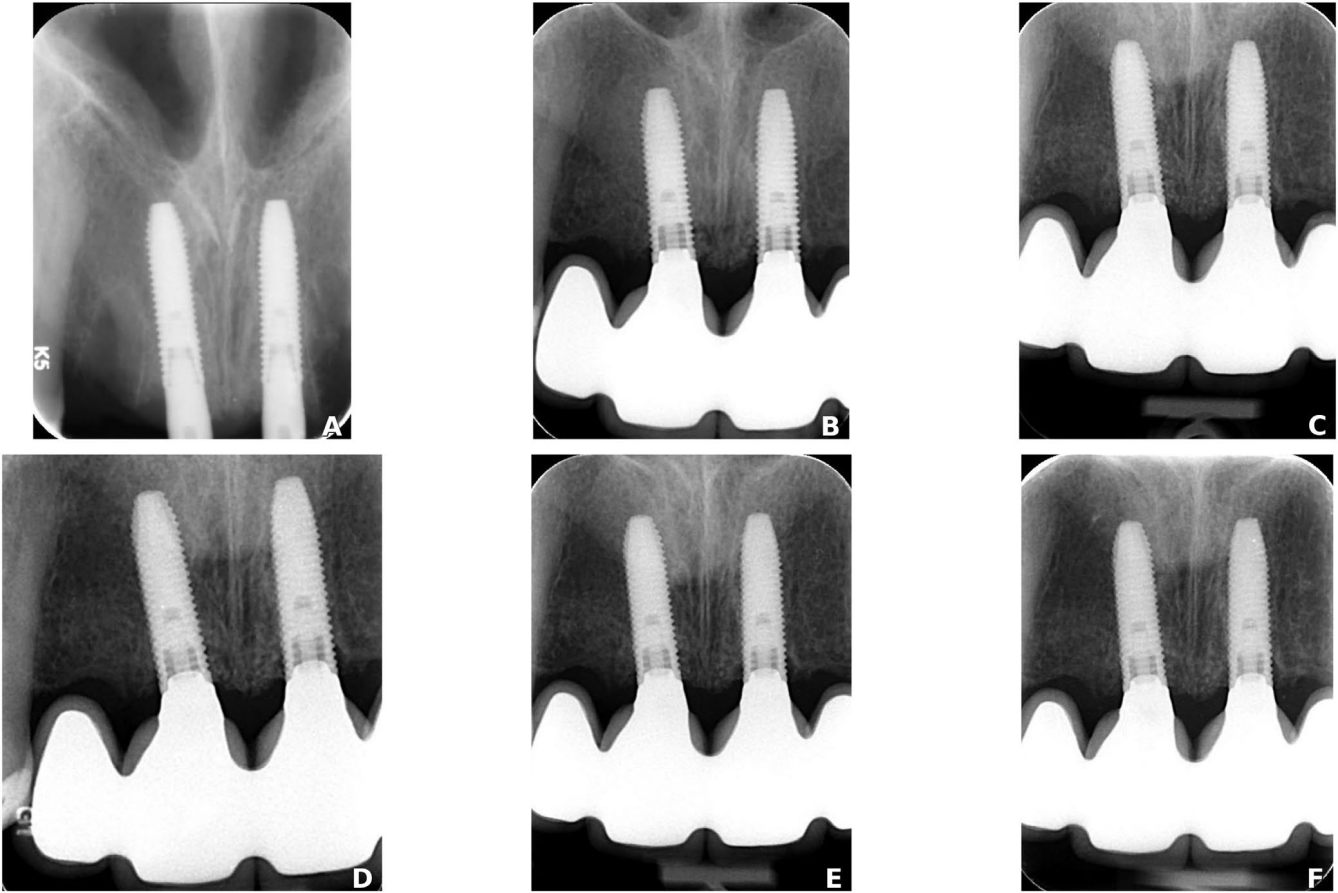
Serial periapical radiographs documenting implant positioning, osseointegration, and marginal bone levels over a 6‐year follow‐up period. (A) Post‐surgical periapical radiograph obtained immediately after implant placement, demonstrating implant positioning and surrounding bone adaptation, obtained on January 25, 2019. (B) One‐year follow‐up periapical radiograph showing satisfactory osseointegration and stable marginal bone levels, obtained on December 16, 2020. (C) Three‐year follow‐up periapical radiograph showing satisfactory osseointegration and stable marginal bone levels, obtained on April 25, 2022. (D) Four‐year follow‐up periapical radiograph showing satisfactory osseointegration and stable marginal bone levels, obtained on January 30, 2023. (E) Five‐year follow‐up periapical radiograph showing satisfactory osseointegration and stable marginal bone levels, obtained on January 16, 2024. (F) Six‐year follow‐up periapical radiograph showing satisfactory osseointegration and stable marginal bone levels, obtained on February 24, 2025.

Three years post‐treatment, the patient requested minor adjustments to the shape of the lateral incisors. The screw‐retained solution allowed easy revision and reinstallation without surgical intervention. At the 6 year follow‐up, the implants remained clinically and radiographically stable, demonstrating favorable long‐term outcomes and maintenance of peri‐implant hard and soft tissues (Figure 9A–F).

## 5 Discussion

MIR presents a substantial clinical challenge because irreversible structural changes may preclude conventional tooth‐preserving treatment [2, 3, 4, 7, 13]. This case illustrates how interdisciplinary collaboration and digital treatment planning can support management in a complex clinical setting.

An interdisciplinary approach was essential for diagnosis, etiological assessment, and treatment planning. Collaboration among general dentists and specialists in endodontics, oral surgery, radiology, and prosthodontics ensured a comprehensive assessment and supported optimal treatment outcomes. Implant therapy was chosen based on prognosis, treatment time, and cost.

Early decision‐making reduced overall costs by avoiding potentially unsuccessful endodontic, restorative, and surgical interventions.

Integration of CBCT, intraoral scanning, and DTX Studio supported accurate and predictable implant planning [8, 9, 10]. Evidence on virtual surgical planning indicates improved accuracy and broad clinical applicability in implant placement workflows [14]. The digital workflow also enabled precise planning of tooth form, position, and emergence profile [10]. In addition, preoperative visualization of the anticipated result increased patient involvement and may have contributed to treatment satisfaction [15].

Digital planning reduced the risk of implant malposition and streamlined the clinical workflow [10, 14]. Immediate loading provided an esthetic and functional restoration on the day of surgery. The screw‐retained prosthesis also allowed straightforward retrieval and later modification, as demonstrated by the revision performed 3 years after treatment without invasive intervention.

Radiographic follow‐up demonstrated stable marginal bone levels with no evidence of peri‐implant bone loss. Furthermore, clinical evaluation revealed no increased probing depths or bleeding on probing, indicating healthy and stable peri‐implant tissue conditions throughout the observation period.

At the 6‐year follow‐up, the implants remained clinically and radiographically stable, and the patient continued to report high satisfaction with both the esthetic and functional outcomes. The screw‐retained solution proved to be a flexible long‐term treatment option, allowing maintenance and future adjustments without invasive procedures.

A limitation of this case report is the absence of objective implant stability measurements such as resonance frequency analysis (ISQ). Primary stability was assessed clinically based on insertion torque and the surgeon's intraoperative judgment and experience.

This case demonstrates that structured interdisciplinary collaboration, supported by digital planning tools, can facilitate successful rehabilitation in a complex case of MIR. The workflow supported precise diagnosis, prosthetically driven implant placement, and individualized restoration planning with active patient involvement. The screw‐retained design also offered long‐term flexibility by allowing maintenance and modification without extensive intervention. Although conclusions from a single case must be interpreted cautiously, this treatment approach may be relevant in similarly complex clinical situations.

## Author Contributions

**Shoresh Afnan:** conceptualization, data curation, formal analysis, resources, software, supervision. **Thomas Alexander Cursiter Hunter:** conceptualization, resources, writing – original draft, writing – review and editing. **Kåre Jan Attramadal:** conceptualization, resources, supervision. **Jashan Singh Kamboj:** conceptualization, data curation, formal analysis, methodology, project administration, resources, software, supervision, validation, visualization, writing – original draft, writing – review and editing.

## Funding

The authors have nothing to report.

## Ethics Statement

Ethical approval was not required for this single case report because no experimental intervention or prospective research involving human subjects was performed. The report describes routine clinical treatment and follow‐up performed as part of standard dental care.

## Consent

Written and verbal informed consent was obtained from the patient for publication of this case report and all accompanying clinical and radiographic images. Consent documentation is retained by the corresponding author and was provided to the journal upon request.

## Conflicts of Interest

The authors declare no conflicts of interest.

## Data Availability

All data are available upon request.
